# Benediction Sign After a Malunited Distal Radius Fracture: A Case Report of Flexor Tendon Rupture Mimicking Median Nerve Injury

**DOI:** 10.7759/cureus.93843

**Published:** 2025-10-04

**Authors:** Xin Yi Nicole Lee, Long Peng Chan, Mala Satku, Sreedharan Sechachalam

**Affiliations:** 1 Yong Loo Lin School of Medicine, National University of Singapore, Lower Kent Ridge, SGP; 2 Department of Hand and Reconstructive Microsurgery, Tan Tock Seng Hospital, Jalan Tan Tock Seng, SGP

**Keywords:** benediction sign, distal radius fracture, drf, flexor tendon rupture, median nerve injury, wrist fracture

## Abstract

The Benediction sign is a pathological hand posture that appears when a person tries to make a fist. It is characterized by the extension of the index and middle fingers, abduction of the thumb, and flexion of the ring and little fingers. Clinically, the appearance of the Benediction sign is closely associated with high median nerve neuropathy. However, in the following case described, a low-energy fall resulted in delayed presentation of a right distal radius fracture together with complete rupture of the flexor digitorum profundus (FDP) tendons to the index and middle fingers and flexor pollicis longus (FPL) within the carpal tunnel. This injury produced a hand posture resembling the Benediction sign, resulting in an initial clinical impression of median nerve injury. Further investigations suggested an alternative diagnosis, raising suspicion of FDP and FPL tendon rupture. Surgical intervention was then undertaken to reduce and fix the malunited distal radius fracture and to repair the ruptured FDP and FPL tendons.

This case emphasizes the need to consider rupture of FDP and FPL tendons as a masquerade of the Benediction sign. We present clinical progression and effective management of this case to underscore the significance of this important diagnostic consideration.

## Introduction

In adults, the most common forearm fractures are distal radius fractures (DRFs), of which the mechanism of injury is most often a fall onto an outstretched hand [1]. General clinical practice accepts conservative management via casting as the first-line treatment for non-displaced DRFs [2]; however, surgical fixation is recommended for displaced DRFs [3]. A meta-analysis by Ochen et al. of 2254 participants demonstrated that adult patients under 60 who underwent surgical treatment of DRFs had better grip strength and medium-term disabilities of the arm, shoulder, and hand questionnaire (DASH) scores, with no increase in overall complication rates [4]. However, the same study revealed no difference in DASH scores for the elderly over 60 who underwent surgical fixation, with higher complication rates, suggesting that conservative treatment would be preferred for the elderly [4].

Reported complication rates of DRFs in the literature range from as low as 6% to as high as 80% [5], with the most common complication being that of malunion of the DRF [6]. Nerve injuries are also a widely reported complication with a prevalence of up to 17% [7], with the median nerve being the most frequently injured [1,8], followed by the radial and ulnar nerves. Median nerve injury typically presents with sensory deficits, pain, and/or paresthesia affecting the thumb, index, middle, and radial half of the ring finger [9]. More proximal injuries may also result in motor impairment involving the pronator teres, flexor carpi radialis, palmaris longus, flexor pollicis longus (FPL), and the lateral portion of the flexor digitorum profundus (FDP) [10]. A characteristic sign of high median nerve injury is the 'Hand of Benediction,' which is a pathological posture seen when attempting to make a fist. It is marked by abduction of the thumb, extension of the index and middle fingers, and flexion of the ring and little fingers [11]. 

A rarer complication of DRFs includes tendon ruptures. If the DRF is managed conservatively, close monitoring of the extensor pollicis longus is required, as it is known to rupture even if the fracture is nondisplaced at a median of seven weeks [12]. If surgical fixation is performed, flexor tendon ruptures may occur as a complication of the surgery [13,14]. In this instance, we describe a unique case whereby a malunited distal radius fracture resulted in ruptures of the FDP and FPL tendons, resulting in a clinical presentation that classically resembled a median nerve injury with the presence of Benediction sign.

## Case presentation

A 63-year-old right-handed female with a history of frequent recurrent falls sustained two low-impact falls onto her right wrist at home over the course of a week in late March 2024. She was admitted to a private hospital on 28th March 2024 as a precautionary measure, where imaging revealed a stable malunion of an old displaced right distal radius fracture. The patient expressed that the injury was likely sustained from a bad fall in December 2023, where she sustained a right wrist injury, for which she did not seek medical attention.

**Figure 1:**
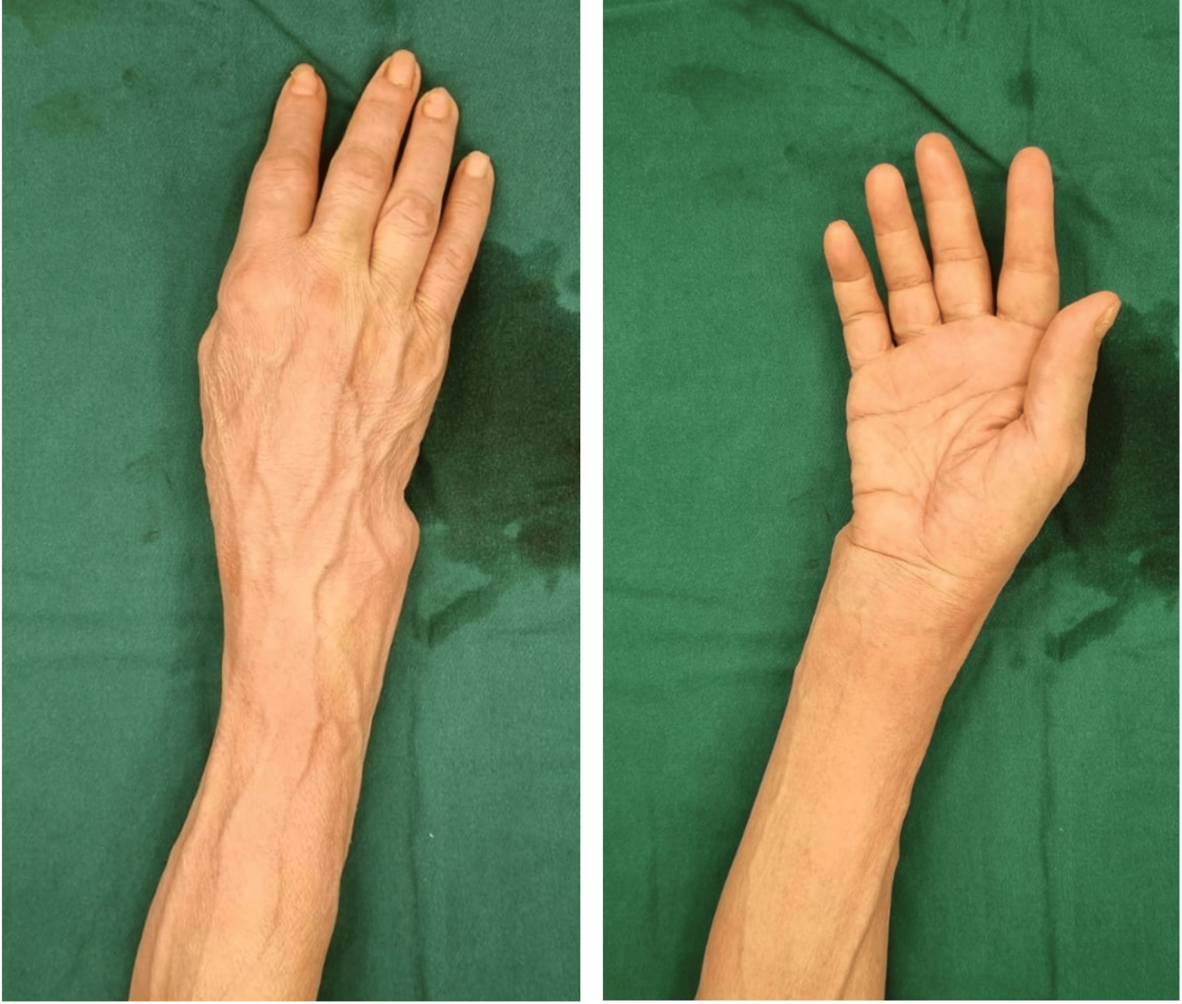
Photographs of the right wrist showing obvious ulnar deformity in dorsal and palmar views, respectively, as well as fading of DIPJ flexion creases in the index, middle, and ring fingers. DIPJ: Distal Interphalangeal Joint

**Figure 2:**
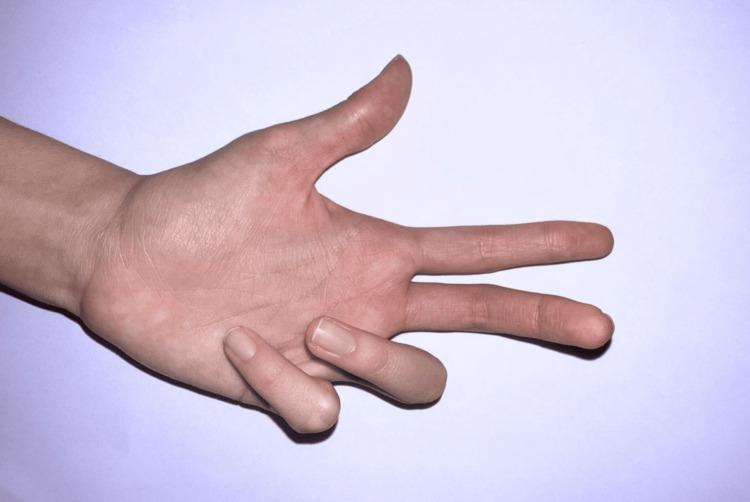
Recreation of ‘Hand of Benediction’ sign. Author's own hand.

On April 2, 2024, the patient presented to our hospital’s Emergency Department for worsening numbness and weakness of three months' duration over the median nerve distribution of the right hand involving the thumb, index, and middle fingers. She had been experiencing numbness in the aforementioned areas for around two to three years; however, the weakness only started after her wrist injury in December 2023. During clinical examination, a visible wrist deformity and evidence of thenar muscle wasting were observed (Figure 1). There was also fading of the flexion creases at the distal (DIPJ) interphalangeal joints of the index, middle, and ring fingers, consistent with a longstanding loss of flexion in these digits. A Hand of Benediction sign was also noted (Figure 2).

**Figure 3:**
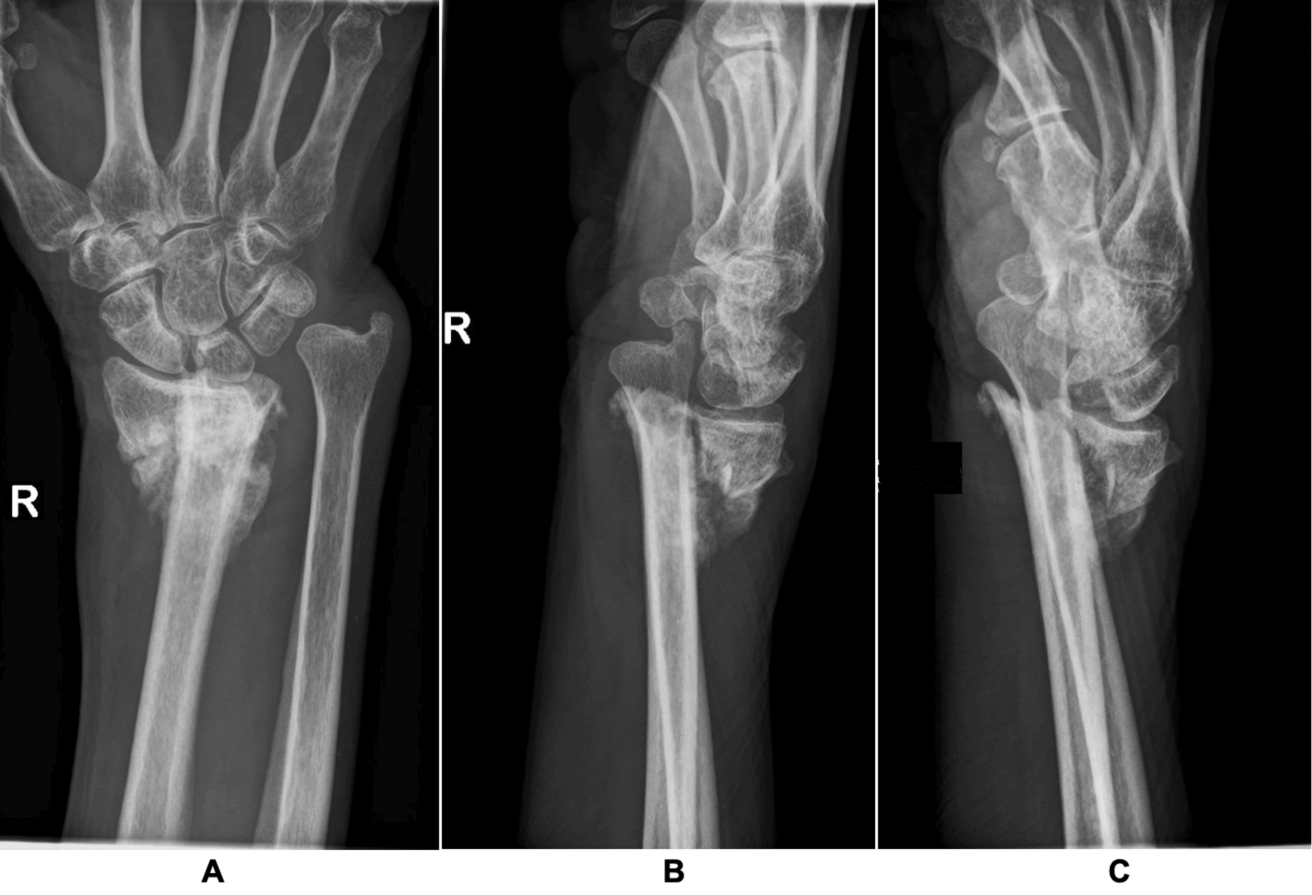
Radiographs of the right wrist (A: posteroanterior view, B: lateral view, C: joint view), showing a dorsally displaced, shortened right distal radius fracture with callus formation.

Sensory testing demonstrated approximately 50% reduction in sensation over the thenar eminence, thumb, index, and middle fingers when compared to the ring and little fingers. Motor assessment showed marked weakness of thumb abduction (MRC 3), complete loss of function (MRC 0) in the FPL tendon to the thumb and FDP tendons to the index and middle fingers, and reduced strength (MRC 4) in the flexor digitorum superficialis (FDS) of the same fingers. All other motor functions in the right hand were intact. Plain radiographs of the right wrist taken at our hospital showed a malunited distal radius fracture with dorsal displacement and shortening with callus formation. Of note, there was a bony prominence at the volar surface of the distal radius (Figure 3).

In view of her presentation of sensory loss and motor weakness in the median nerve distribution of the right hand, along with the presence of a Hand of Benediction sign, the clinical impression was of median nerve neuropathy caused by the malunion of her distal radius fracture. While her weakness in finger flexion could also be attributed to flexor tendon ruptures, such injuries are exceedingly rare following distal radius fractures and are typically only reported as complications of surgical fixation. As the patient had not undergone any surgical intervention and also exhibited sensory loss in the median nerve distribution, median nerve neuropathy was considered the primary differential diagnosis.

Nerve conduction studies (NCS), electromyography (EMG), and ultrasound (US) studies of the right median nerve were performed. However, the investigations yielded surprising results. While NCS demonstrated findings consistent with mild to moderate right carpal tunnel syndrome, EMG results were within normal limits. Furthermore, US imaging revealed a normal cross-sectional area of the median nerve at the region of interest. Therefore, the findings suggested that median nerve neuropathy was unlikely to be the primary cause of her symptoms.

In light of these findings, it was considered that the patient’s FPL and FDP tendons to the right index and middle fingers may have been ruptured secondary to the malunited distal radius fracture, thereby mimicking a Benediction sign suggesting median nerve injury. Subsequently, the patient was counselled for surgical intervention. 

**Figure 4:**
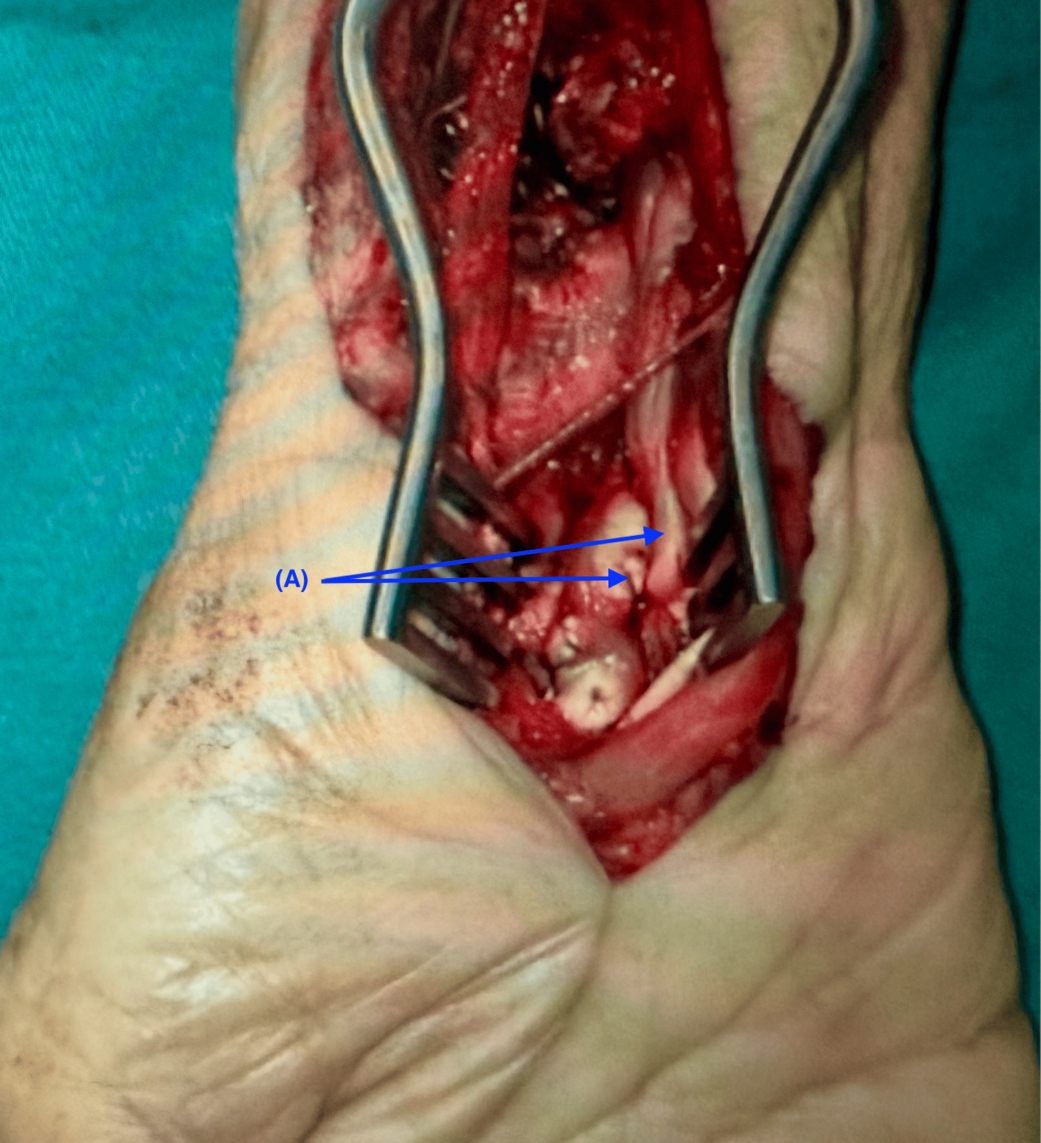
Intraoperative picture showing ruptured tendons in situ. Blue arrow (A) shows the site of ruptured tendons.

**Figure 5:**
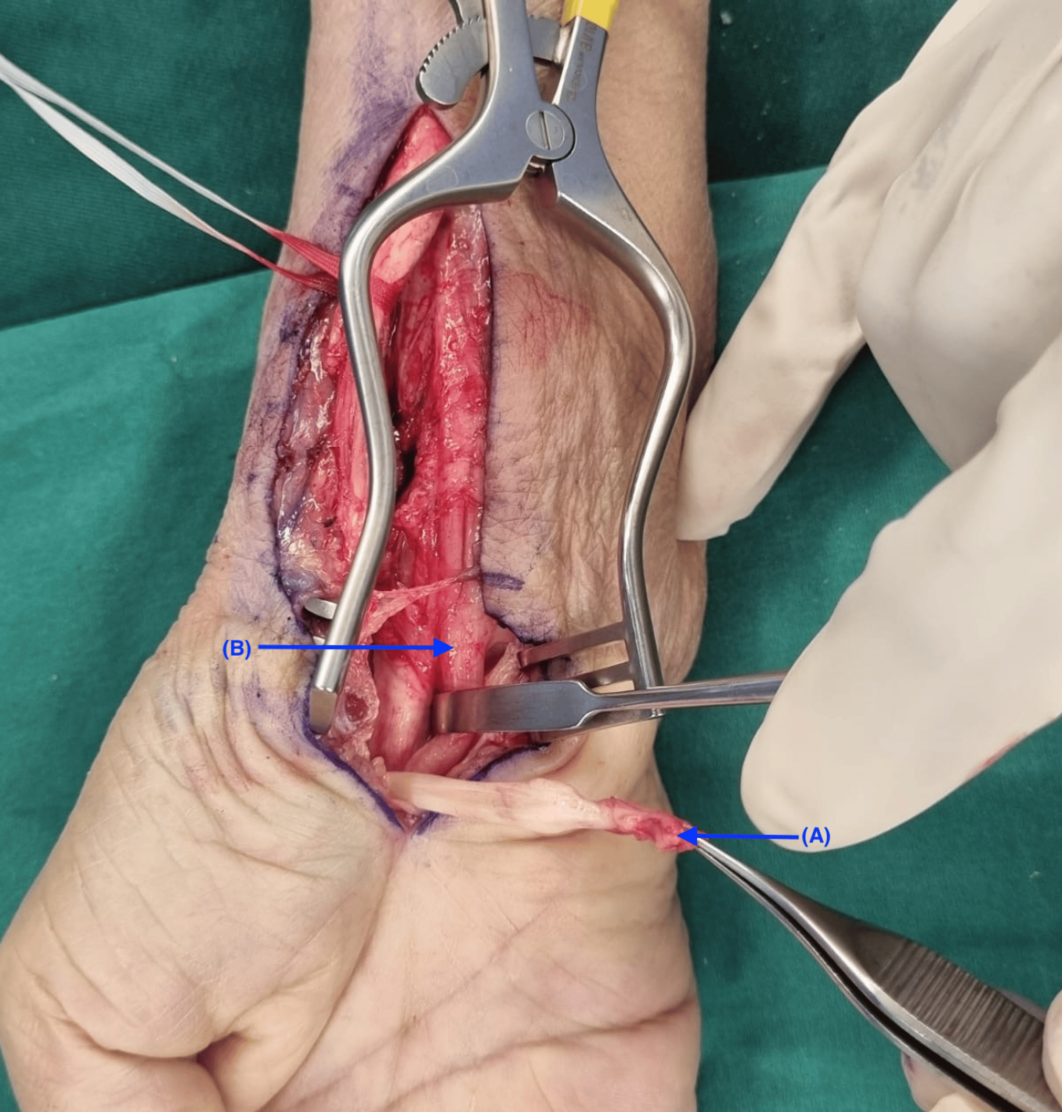
Intraoperative picture showing ruptured flexor tendons. Blue arrow (A) indicates ruptured tendon. Blue arrow (B) shows the median nerve in continuity.

Three weeks after the initial presentation, right distal radius malunion with distal fracture fragment and dorsal carpus dislocation was identified during surgical exploration. In addition, the FPL and the FDP tendons to the index and middle fingers were found to be completely transected within the carpal tunnel (Figures 4, 5). The patient underwent right distal radius corrective osteotomy, carpal tunnel release, distal ulna osteotomy, and tendon grafting of the index and middle finger FDP tendons to the ring finger FDP tendon. 

**Figure 6:**
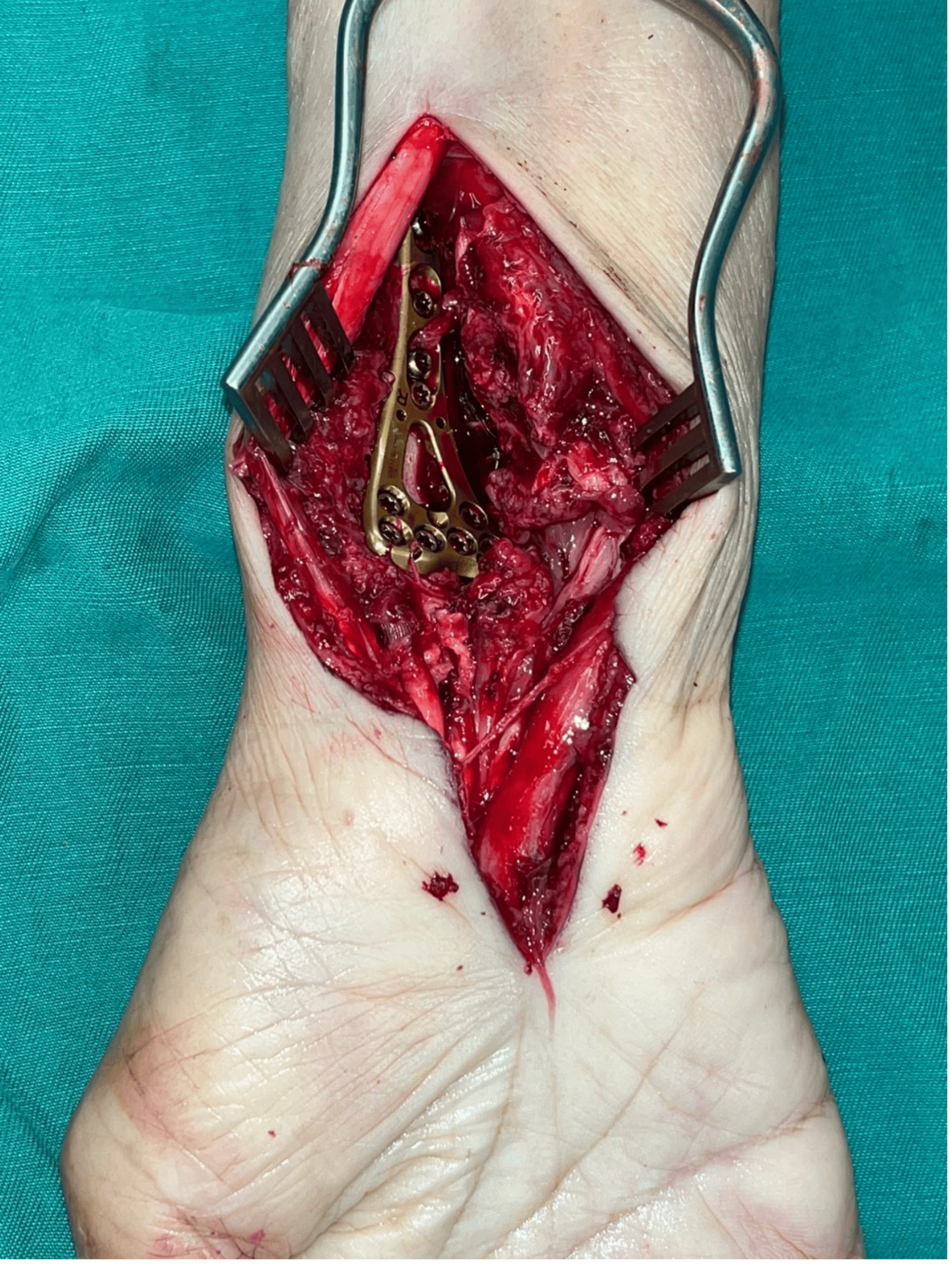
Intraoperative picture showing fixation of the distal radius in a more anatomical position.

**Figure 7:**
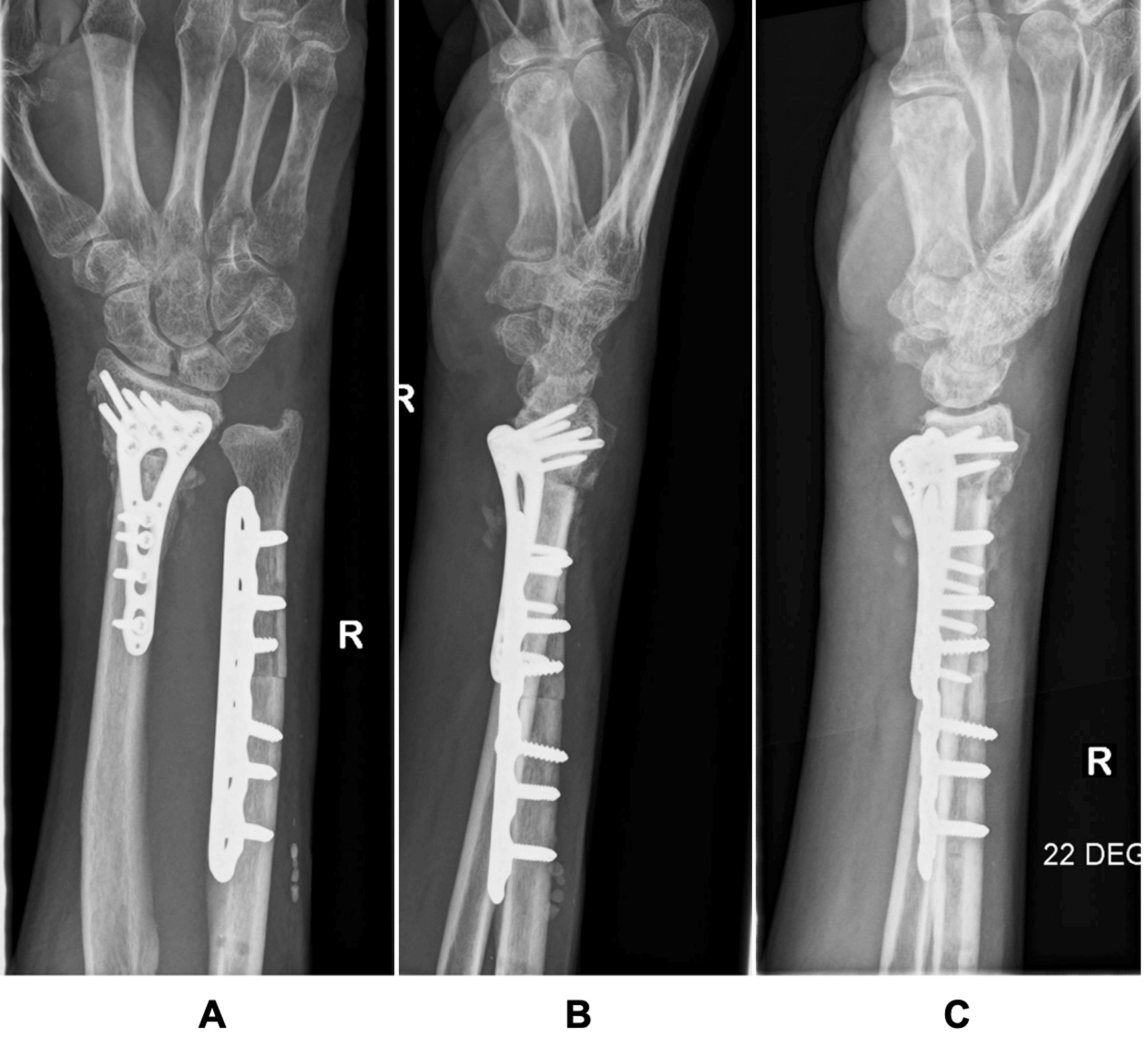
Post-operative radiographs (A: posteroanterior view, B: lateral view, C: joint view) of the right wrist showing correction of distal radius malunion and ulna shortening.

The distal radius was shortened before the fracture was reduced and fixed with a 4-hole 2.4 mm distal radius Variable Angle Locking Compression Plate and screws (DePuy, Synthes, West Chester, PA) (Figure 6). To correct the persistent positive ulnar variance, a distal osteotomy was performed with 15 mm of ulnar bone excised and fixed with a 3.5 mm Locking Compression Plate (DePuy, Synthes, West Chester, PA). To address the transected tendons, adhesiolysis of the FDP tendons to the index and middle fingers was first performed. The FDP of the index finger was then secured directly to the ring finger FDP using a Pulvertaft weave and secured with PDS 3-0 and a running continuous suture. Subsequently, the FDP to the middle finger was also secured to the ring finger FDP, similarly using PDS 3-0 and a running continuous suture. At the surgeons’ discretion, the ruptured FPL was not repaired, as the patient had sufficient premorbid thumb metacarpophalangeal joint (MCPJ) flexion, allowing adequate key pinch and grasp positions of the thumb. Radiographs showing surgical correction of distal radius malunion and ulna shortening are provided below (Figure 7).

**Figure 8:**
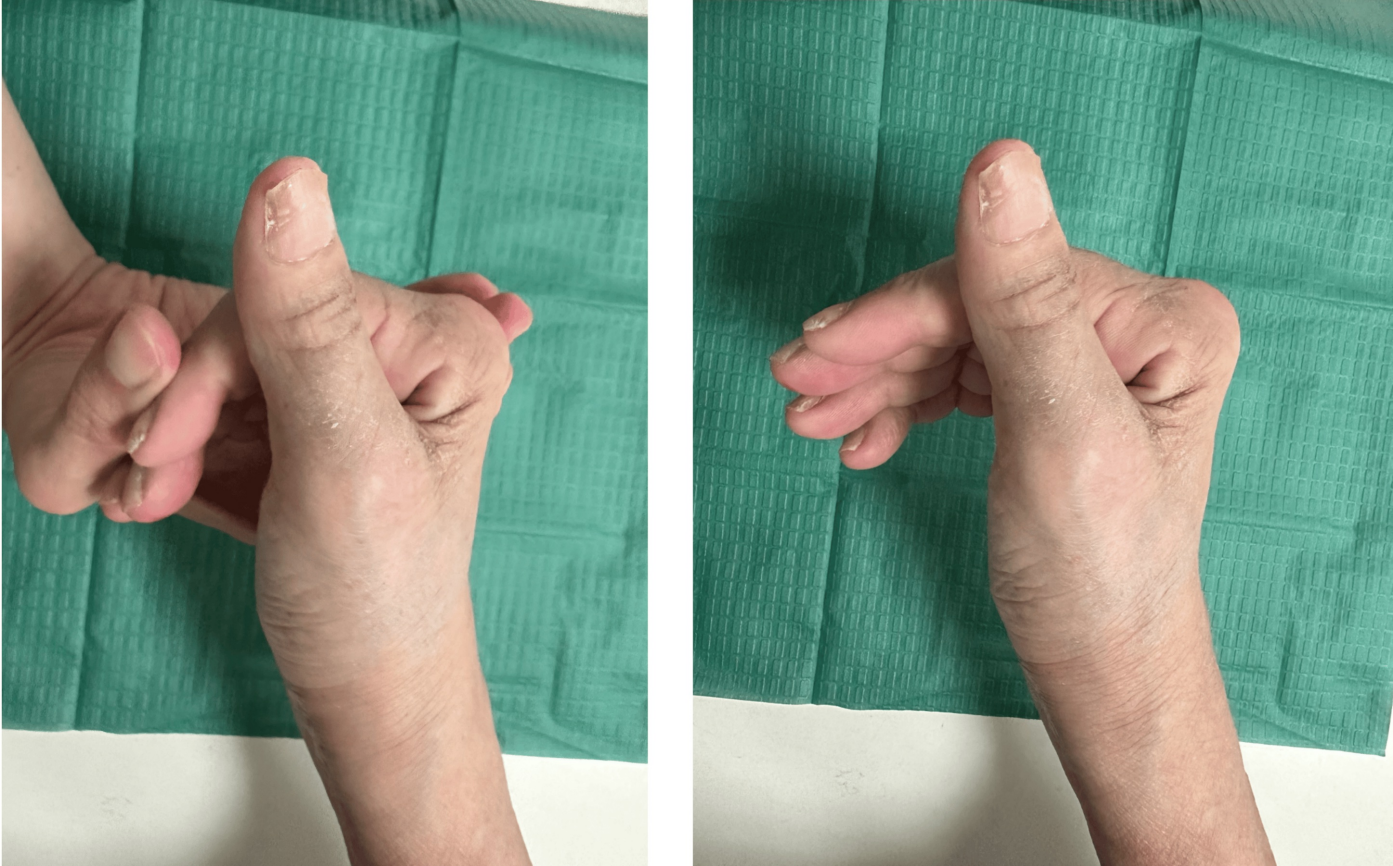
Photographs showing passive and active range of motion, respectively, at postoperative week six.

Post-operatively, the patient underwent a course of eight sessions of occupational therapy (OT) weekly for two months and defaulted on follow-up thereafter. The repair was strong enough to tolerate early active movement. By post-op day two, the patient was able to flex the DIPJ of her index and middle fingers. By post-op week one, the patient was able to actively flex all fingers, with passive flexion to about 50% fist and active flexion of the index and middle finger DIPJ to 35°. At one month post-op, wounds were noted to be well healed, and the patient could actively flex to form a 50% fist (Figure 8). She had also regained enough strength to lift a cup with water off the table via key pinching of the cup handle. At six weeks post-op, the patient was able to flex the thumb interphalangeal joint (IPJ) to 25°, the index finger proximal interphalangeal joint (PIPJ) to 65°, and the middle finger PIPJ to 60°. She was then discharged from the community hospital.

However, she was non-compliant with her physiotherapy regimen and subsequently was unable to maintain her DIPJ function, likely due to the formation of adhesions. As such, her last recorded DIPJ range of motion was recorded at 0-10° for her right index finger and 0-35° for her right middle finger at approximately 15 months postoperatively, despite being able to achieve better range of motion in the initial days post-surgery. She was able to carry out her activities of daily living without any issues and reports being able to open a tight jar and use a knife to cut food easily with her right hand. 

## Discussion

Although uncommon, delayed flexor tendon ruptures have been reported in the literature following nonoperative management of distal radius malunions [15]. Two primary mechanisms have been proposed for these ruptures [16]: 1) acute transection of the tendons by fracture fragments and 2) chronic attritional damage caused by bone spurs or anterior bony prominences of the distal radius [17] and/or volar displacement of the ulnar head [18]. In our case, we believe that tendon ruptures of the FDP and FPL in our patients likely resulted from chronic attrition; however, it is difficult for us to conclude this, as the patient only sought medical attention months after the initial injury. 

A detailed history and physical examination is particularly important in our case, as the clinical signs of numbness and weakness of flexion of the thumb, index, and middle fingers closely mimicked the presentation of median nerve neuropathy. Additionally, given that nerve injury is a much more common complication of distal radius fractures, the initial clinical impression was of median nerve injury. Standard investigations for median nerve neuropathy, such as NCS, EMG, and US of the median nerve, were performed to check for median nerve etiology, and the results pointed us towards a tendon pathology instead.

In managing such cases, the literature emphasizes two critical components: correction of the distal radius malunion and reconstruction of the affected flexor tendons. In our case, a corrective osteotomy was performed to realign the distal radius, and the ruptured FDP tendons to the index and middle fingers were grafted onto the FDP of the ring finger. As noted by Komura et al. [15], both tendon transfer and tendon graft techniques have shown comparable outcomes, with no consensus on which approach is superior for reconstruction. During the surgery, a distal ulna osteotomy was also performed to address the positive ulnar variance, which had been further accentuated by the distal radius shortening following the osteotomy.

## Conclusions

In conclusion, this case report highlights a case of delayed flexor tendon ruptures following a dorsally displaced distal radius fracture, with a clinical presentation that closely resembled median nerve pathology. Earlier recognition and timely management of the fracture might have reduced the risk of tendon rupture. In cases where tendon rupture occurs after nonoperative treatment of a distal radius fracture, surgical correction of the bony deformity and tendon reconstruction should be performed to restore function and prevent further complications.
